# Social cognition and emotional rehabilitation in participants with schizofrenia

**DOI:** 10.3389/fpsyt.2023.1250933

**Published:** 2023-11-08

**Authors:** Francisco Rodríguez Pulido, Nayra Caballero Estebaranz, Alejandro Alberto García Caballero, Enrique González Dávila, Celia León Palacín, María del Carmen Hernández Álvarez de Sotomayor, Susana López Reig, Patricia Inés Vílchez de León

**Affiliations:** ^1^Department of Internal Medicine, Dermatology and Psychiatry, University of La Laguna, San Cristóbal de La Laguna, Spain; ^2^Department of Health Sciences, European University of the Canary Islands, La Orotava, Spain; ^3^IPS Team Sinpromi, Cabildo de Tenerife, Santa Cruz de Tenerife, Spain; ^4^Complexo Hospitalario Universitario de Ourense day hospital, Orense, Spain; ^5^Department of Mathematics, Statistics and Operations Research Department, University of La Laguna, San Cristóbal de La Laguna, Spain; ^6^Canary Islands Association of Creative Therapies (ASCATEC), Santa Cruz de Tenerife, Spain

**Keywords:** cognition, emotional perception, schizophrenia, theory of mind, employment

## Abstract

**Introduction:**

People with schizophrenia have deficits in social cognition, emotion and social perception, as well as attributional style. The purpose of this study was to evaluate the efficacy of a multicomponent social cognition training program, e-Motional Training® (ET), in people with schizophrenia and to compare its efficacy with people who did not receive it. Therefore, a single-blind RCT was conducted in participants with a diagnosis of schizophrenia.

**Methods:**

A randomized, single-blind, clinical trial was conducted with 50 stable outparticipants with schizophrenia (registry number CHUC_2019_109). All participants (control and intervention) were treated with pharmacotherapy, case management and were on Individual Placement and Support methodology for competitive employment. The intervention group was treated with ET, an online program designed for social cognition rehabilitation. Pre and post assessment was performed using different battery of tests. General mixed models with subject identification and repeated measures over time were used.

**Results:**

Different pre and post measurements were performed in the two groups. No significant differences were found in sociodemographic characteristics between the control and intervention groups. Improvements were obtained in the intervention group in the Ekman test (*p* = 0.009), mainly enhanced by the improvement shown in three emotions: fear, sadness and disgust (*p* = 0.041, *p* = 0.021 and *p* = 0.038 respectively).

**Conclusion:**

ET is a promising online training tool for social cognition deficits in schizophrenia, in particular, for the improvement of emotions.

**Clinical Trial Registration**: https://beta.clinicaltrials.gov, NCT05866328.

## 1. Introduction

Schizophrenia is a mental disorder that has as one of its distinctive features the presence of difficulties associated with poorer social functioning (1), fewer social relationships, and poorer quality of life (2, 3). People with schizophrenia often become socially isolated, showing less initiative and motivation toward activities, especially social activities, than they did before the onset of the illness. They suffer from social isolation because they find it difficult to make contact with other people and to maintain a conversation. They have difficulty functioning normally under stressful conditions, partly because of their difficulties in social skills and problem solving (4, 5).

Couture et al. (6) concluded that there are clear and consistent relationships between aspects of functional outcome and social cognition. However, methodological issues remain unresolved (7–9) on the recognition of facial affect as a mediator between cognitive and social functioning in psychosis.

Since the 2000s, interest in Social Cognition (SC) has been growing after it was shown that participants with Severe Mental Disorder (SMD) with problems in this type of tasks presented worse social functioning (3) and poorer social relationships (10). SC includes the perception of emotions, both in people’s faces and voices (11, 12), social perception (6, 13) and life perception (14–16). During the last decade, there have been improvements on the development of rehabilitation tools focused on SC which have proven that these deficits are restorable and that their improvement contributes to functional recovery (17–21), theory of mind (ToM) (22, 23) and attributional style (AS) (24–27).

Different work teams have suggested an alternative to group training using computerized training which would allow virtually universal accessibility (28). e-Motional Training® (ET) is an online self-training program for the SC rehabilitation of participants with SMD such as schizophrenia. This program was initially developed and tested by the Psychiatry Service of the Complexo Hospitalario de Ourense. The first version of the program started developing in 2011, being operational in 2013. The results of the initial unblinded controlled pilot study demonstrated statistically significant improvements in both Emotion Recognition (ER) and ToM (29). The version 1.0 has proven its efficacy in a multicenter randomized clinical trial (RCT) (30) and version 2.0 of the program, developed since 2013, is now available (31). ET 2.0 increased the number of different models with photographs, the number of games and a new movie was included with actors called “The Surprise.” In addition, a first prosodic training game VOICES, a mime training game and a video game for ToM (Second Chance) was included.

Currently there are some SC training programs that have reported promising results in cognitive and functional outcomes (32, 33). These encouraging results are limited in a practical sense because their application requires complex clinical settings, with specially trained and skilled medical teams for their implementation, resulting in higher costs that limit their accessibility to the entire target population. ET arises from this need, with the objective of developing an easy to apply tool in Spanish, with the possibility of being self-administered by the patient and that is also versatile for the rehabilitation of social cognition.

Employment is another concern that has become apparent in the scientific literature in recent years (34). The high unemployment rates of people with schizophrenia have led to strategies that have been systematically evaluated. One of these strategies is Individual Placement and Support (IPS), developed in the US by Becker and Drake (35), which establishes that incorporation into competitive employment should occur as quickly as possible, followed by support and training in the job. The person with SMD must be integrated into the workplace in the same way as another person without a mental disorder, with the purpose of emphasizing the social integration of the patient, reinforcing the significance of his role as a citizen and making him an essential participant in his therapeutic recovery process (36). There is strong evidence, both in Europe and in the US, that the IPS model is more effective for people with SMD to obtain competitive employment than programs based on pre-vocational training such as the “Train-Place” (37, 38). In addition, the IPS presents better results than pre-vocational training, considering both the number of hours and weeks worked competitively, and the number of hospital admissions (36–38).

The focus of this study is to analyze the impact of the ET intervention on social competence in people with schizophrenia who use the IPS employment strategy.

## 2. Materials and methods

### 2.1. Sample size

**Figure 1:**
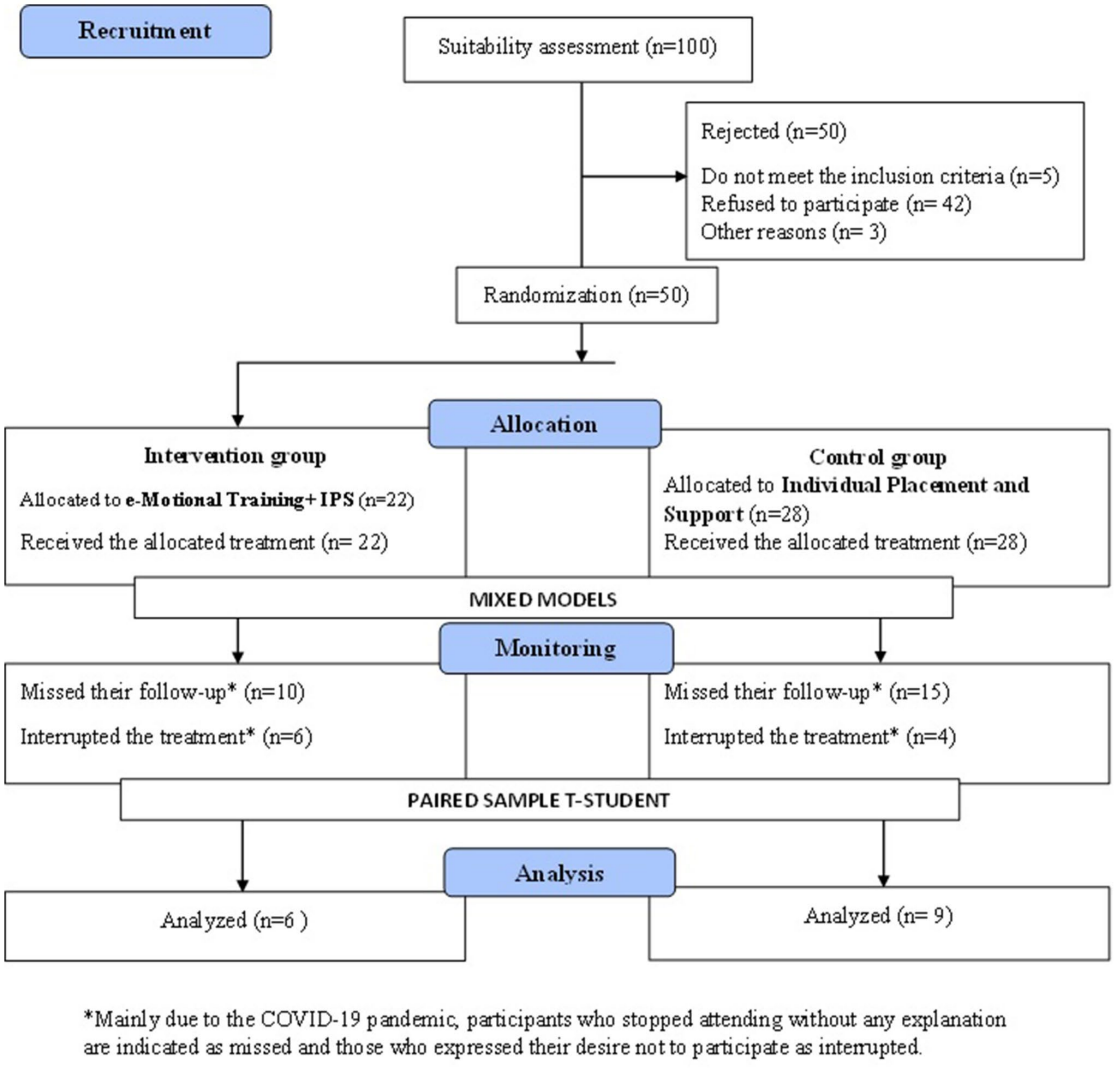
consort flow diagram.

A randomized single-blind clinical trial was conducted and participants in this study were recruited from the Community Mental Health Units (USMC in Spain) and basic health areas on the island of Tenerife, Canary Islands. A total of 100 participants were recruited, of which 50 were excluded because 5 did not meet the inclusion criteria, 42 refused to participate and 3 were discarded due to other reasons. The other 50 participants were randomized and randomly assigned to the control group (28 persons, 56%) and to the intervention group (22 persons, 44%). During follow-up, 19 users were lost in the control group and 12 in the intervention group, and 4 discontinued the treatment intervention. At the end, only a total of 6 participants belonging to the intervention group and 9 to the control group presented information from both periods (Figure 1). A power study was carried out considering that the target variables (emotion recognition) were defined as the difference between the post and pre value, setting a precision to be detected of a standard deviation between the control and intervention group and with a confidence level of 95%. In the case of the initial sample size of 50 participants (assuming a 10% loss to follow-up), the statistical power would be 90%, while with the sample size of patients with both tests, *N* = 15, this would drop to 41.2%. All participants gave both verbal and written consent to participate in this study.

### 2.2. Inclusion and exclusion criteria

The patients included voluntarily agreed to participate in the study, aged 18–50 years with a diagnosis of schizophrenia (DSM-V criteria), who were clinically stable (no acute psychotic symptoms and not hospitalized during the last 3 months) able to consent to their voluntary participation in the study. Exclusion criteria were considered, in addition to not meeting the inclusion criteria, having a comorbid SMD or to present a history of severe brain damage or neurological disorder that could act as a confounding factor, or intellectual disability (such as associated disorder of organic type or diagnosis of borderline or lower IQ), participate in another skills program aimed at improving social adjustment and manifest active substance abuse (except nicotine). Participants who explicitly indicated a desire to terminate participation in the study were withdrawn.

### 2.3. Treatment conditions

The duration of the study was expected to be 1 year, but due to the worldwide pandemic of COVID-19, it was extended to 2 years. All tests were performed during morning working hours at the Island Society for the Promotion of People with Disabilities (SINPROMI). The study participants were randomly distributed in:

- Intervention group: Employment support intervention using the IPS strategy together with cognitive rehabilitation through the ET program.
- Control group: Employment support treatment using the IPS strategy.

The intervention group received the same intervention as the control group plus 12 sessions with Emotional Training®. All participants in the intervention group completed the same number of sessions. To begin the intervention, the patient accessed the website^1^ (version 2.0) and registered with a username and password. The first four meetings (1 h each session) were dedicated to recognizing facial emotions. This section included a pretest and a posttest, tutorials, and scaling minigames starting with eyes and mouths and finally microexpressions.

Both groups underwent a battery of tests before and after treatment (these tests will be discussed in more detail in the following section). This battery of tests was performed by a blinded researcher, psychologist specializing in treatment with schizophrenic patients (the same for all participants), in 3 sessions of 1 h each in order to assess the baseline level of social cognition and determine whether the intervention was advantageous or not.

The standard treatment group (control group) initially received the pre-assessment sessions at SINPROMI and after 4–5 months the users were contacted again for post-testing at the same place. During this process, all participants continued to perform the normal activities carried out in this center and had access to job offers.

ET is designed following the basic principles of neuropsychological rehabilitation in this domain (39–41). ET 2.0 have improvement to ET 1.0 version. Its primary objective is to offer realistic and natural exercises that are engaging and short in duration without irrelevant stimuli or distractions, while providing continuous feedback. The program consists of two modules. The first module is called “Emotions” and deals with emotional perception training, including emotion games with faces, psycho-educational tutorials with static and dynamic images, micro-expression recognition training and initial and final evaluation tests where the participant can observe his or her progress. And the second module is designated “The Party?” whose objective is to train other SC components such as ToM and social perception through the visualization of a short cartoon film, offering automatic metacognitive feedback on their responses. ET includes the following training strategies: continuous feedback automatic metacognitive feedback, repetitive practice, virtual coaching, and continuous assessment [For more information about the social cognition training program, visit (see text footnote 1) (42)].

The intervention group, in addition to the pre and post assessments, attended a weekly session lasting 1 h and 30 min for a total of 3 months. Additionally, one more month was included to recover those sessions that could not be held due to attendance problems, mainly due to the effect of COVID-19. During the intervention, a member of the research team was present at all times to resolve any doubts that might arise. The training program consisted roughly in the individual practice of tasks related to social skills using an interactive video game on computer. In order to start the treatment, the patient had to register with a username and password on the website (see text footnote 1). The intervention program consisted of a total of 12 sessions that were divided into two parts: the first 4 sessions were focused on the first module and the following 8 sessions on the second module. Once the program was completed, the results of each session were stored individually in a database with restricted access to the research staff.

### 2.4. Measuring tools

The independent clinical health psychologist in charge of assessing the measurements did not know whether the person had received treatment or belonged to the control group. The pre and post assessment was performed using the following battery of tests in both groups.

#### 2.4.1. Ekman 60 faces test (43)

A computer test that assesses emotional processing through facial recognition of emotional expressions. It contains 60 photographs of people’s faces with expressions of the 6 basic emotions (joy, fear, surprise, sadness, disgust, and anger). An overall score of 60 indicates the best possible performance, and each basic emotion also has a maximum score of 10 points.

#### 2.4.2. Hinting task (44)

This test is intended to assess ToM by means of 10 short stories. The participant must identify in each story what one character wants to tell the other, measuring the test, the ability to infer the real intention of the character. If the subject answers the question correctly, it is scored with a 2 (Total 1). If not, information is added that makes the hint more clearly identified. If the subject answers correctly, then it is scored 1 (Total 2). A high Total 1 or low Total 2 score will be indicative of a better status.

#### 2.4.3. Happé’s strange stories test (45)

A test that attempts to assess theory of mind by means of 16 stories. Half of the stories require the use of the ToM for their comprehension and the other half do not (control). It determines the ability to deduce mental states referred to desires, beliefs or intentions of some character, including lies, ironies and white lies. To obtain good results in this test, the attribution of mental states such as desires, beliefs, intentions and complex mental states is required.

#### 2.4.4. Faux pas recognition test (46)

This test evaluates ToM through 20 stories. Half of the stories contain a gaffe and the other half contain a minor conflict (control). It measures the ability to detect the understanding of figurative meanings when someone says something inappropriate but without malicious intent. The test provides scores for five variables: faux pas detection, understanding inappropriateness, intentions, belief, and empathy.

#### 2.4.5. Ambiguous intentions hostility questionnaire [AIHQ] (47)

This questionnaire assesses causal attribution through 15 hypothetical situations that can be ambiguous, intentional, or accidental. It explores the attributional cognitive biases (hostility, intention, anger, guilt and aggressiveness) based on the characters’ intentions. The AIHQ yielded hostility perception and aggressive response bias scores and a composite blame bias score. The scales for the hostility perception and aggressive response indices were rated by the rater from 1 (“not at all hostile”) to 5 (“very hostile”) and from 1 (“not at all aggressive”) to 5 (“very aggressive”), respectively. The composite blame score (range 1–5.3) is an average score of subjects’ ratings of intent (range 1–6; rating about the degree to which the other person committed the act on purpose), anger (range 1–5; rating about how angry the situation would make subject feel), and blame (range 1–5; score on the degree to which subjects blame the other person for the outcome).

#### 2.4.6. Movie for the assessment of social cognition [MASC] (48)

This is a social cognition tool that assesses the understanding of nonverbal communication, irony, sarcasm, implicit social rules, blunders or gaffes, and innuendoes. It consists of watching a movie where the participants must understand the various interactions that are established between the characters, answering 50 multiple-choice questions about the emotions, thoughts and intentions of the protagonists.

#### 2.4.7. Screening for cognitive impairment in psychiatry [SCIP-S] (49)

A test that detects the main cognitive deficits that people with mental illness may present. It consists of 5 short tests that explore memory, attention, executive function and processing speed.

#### 2.4.8. Brief symptom checklist [BSL-50] (50)

It identifies and assesses psychological and psychosomatic symptoms in adults. It consists of 7 main scales (obsessive sensitivity, anxiety, hostility, somatization, depression, strict sleep and extended sleep), 2 subscales (sensitivity and obsession-compulsion) and 1 scale (psychopathological risk).

#### 2.4.9. Positive and negative symptom scale [PANSS] (51)

It consists of 30 items that assess schizophrenic disorder from a dual perspective. The first is dimensional and estimates the severity of the positive, negative and general psychopathology syndrome, while the second classifies it as positive, negative or mixed.

### 2.5. Statistical analysis

For the control group and the intervention group, a battery of different tests was executed both pre- and post-intervention. Generalized linear mixed models (with identity link function except the logit link for the variable MASC Success C) with subject identification and with repeated measures over time (before and after) were used, taking into account that all participants do not have pre and post information. The data from both groups were analyzed jointly, examining the group factor to show differences between control and intervention, the time factor to verify differences between pre and post and the interaction between time and group to reflect whether the evolution over time varies according to the group to which one belongs. Additionally, the analysis within each of the groups independently was also included using paired sample t-student considering individuals with pre and post values, as well as independent samples t-student to analyze the pre results between both groups. Holm-Bonferroni correction was applied for multiple comparisons. The data were analyzed with SPSS 26.0 software and differences were considered statistically significant at *p* < 0.05.

## 3. Results

All participants had a diagnosis of schizophrenia, with a mean age of 44.1 ± 6.7 years. Twenty-four percent were female to 76% who were male. The marital status of most of the sample components, 94%, was single or divorced. The educational level of 48% of the participants was primary school, 20% were secondary school, 18% had higher education, 8% were high school graduates and 6% were university graduates. At the beginning of the study, 64% of the participants were unemployed and 36% were working. Mean duration of schizophrenia diagnosis of all participants was 20.8 ± 7.9 years. Finally, 36% of participants had not had any admission prior to the resource, 36% had been admitted once, 20% had been admitted twice and 8% had been admitted three or more times. Regarding the data related to the sociodemographic characteristics of the participants (Table 1A), no significant differences were found between the control group and the intervention group, thus evidencing that both groups are quite homogeneous at the sociodemographic level due to the characteristics related in this regard.

**Table 1A:** Demographic and clinical characteristics of the sample (*N* = 50).

	Control (*N* = 28)	Intervention (*N* = 22)	Global (*N* = 50)	*p* value
Age (years)	44.2 ± 6.2	43.9 ± 7.4	44.1 ± 6.7	0.842
Sex *n* (%)				0.186
Men	19 (68)	19 (86)	38 (76)	
Women	9 (32)	3 (14)	12 (24)	
Marital status				0.246
Single/divorced	25 (89)	22 (100)	47 (94)	
Married	3 (11)	-	3 (6)	
Level of education				0.515
Primary	12 (43)	12 (54)	24 (48)	
Secondary	7 (25)	3 (14)	10 (20)	
Vocational training	6 (21)	3 (14)	9 (18)	
High School	1 (4)	3 (14)	4 (8)	
University degree	2 (7)	1 (4)	3 (6)	
Occupation at the beginning of the study				0.249
Unemployed	20 (71)	12 (54)	32 (64)	
Employed	8 (29)	10 (46)	18 (36)	
Years with illness (years)	20.1 ± 7.4	21.8 ± 8.6	20.8 ± 7.9	0.446
Number of hospital admissions*				
Prior the resource	1 (0; 1)	1 (0; 2)	1 (0; 2)	0.194
After 2/11/2019	0 (0; 0)	0 (0; 0)	0 (0; 0)	0.375
Number of new jobs*	0 (0; 1)	1 (0; 2)	1 (0; 1)	0.239

Data shows mean ± standard deviation or frequency (percentage), except * median (range interquartile).

The comparison of the pre-values between participants who continue the study (carry out the post-test) and those who do not, is included in Table 1B. No significant differences were observed between the variances of these two groups in any of the variables evaluated. Nor are significant differences observed in the means, except for the emotion Fear, with a slight deterioration in the group that does not continue (*p* = 0.049) and, in Hinting Total 1, with a slight improvement in the group that does not continue (*p* = 0.040).

**Table 1B:** Comparison of pre values between participants who continued the study and those who did not.

	Participants who:		*p*-values	
	continue the study (*N* = 15)	did not continue (*N* = 35)	Variance comparison	Means comparison
Ekman Total	47.93 ± 6.05	46.06 ± 6.96	0.660	0.369
Happiness	9.93 ± 0.26	9.60 ± 1.54	0.143	0.411
Surprise	9.33 ± 0.90	9.09 ± 1.79	0.478	0.614
Fear	6.67 ± 2.13	5.20 ± 2.44	0.508	0.049
Sadness	7.40 ± 2.23	8.00 ± 1.96	0.126	0.345
Disgust	6.87 ± 2.23	6.63 ± 2.45	0.626	0.748
Anger	7.73 ± 1.58	7.43 ± 2.03	0.328	0.608
Hinting total 1	12.80 ± 4.89	15.39 ± 3.45	0.195	0.040
Hinting total 2	2.33 ± 1.84	1.39 ± 1.34	0.450	0.052
Happé C	9.20 ± 2.81	8.38 ± 2.68	0.696	0.349
Happé ToM	9.93 ± 3.41	9.88 ± 3.25	0.867	0.958
Faux pas FP-FP	31.87 ± 15.17	33.35 ± 10.81	0.177	0.727
Faux pas C-FP	18.27 ± 1.87	16.96 ± 4.13	0.062	0.258
Faux pas FP-C	18.80 ± 1.21	18.35 ± 1.75	0.646	0.388
Faux pas C-C	18.53 ± 1.51	17.96 ± 1.46	0.357	0.247
AIHQ HB	1.55 ± 0.26	1.54 ± 0.22	0.704	0.909
AIHQ IS	3.14 ± 0.60	3.13 ± 0.90	0.214	0.969
AIHQ AS	2.50 ± 0.64	2.54 ± 0.77	0.222	0.852
AIHQ BS	2.70 ± 0.67	2.57 ± 0.71	0.996	0.581
AIHQ AB	1.44 ± 0.25	1.53 ± 0.25	0.975	0.309
MASC Hits	24.80 ± 6.78	24.90 ± 6.73	0.950	0.966
MASC Success C*	10 (67%)	13 (65%)		0.921
MASC No ToM	4.67 ± 3.15	4.50 ± 3.25	0.765	0.880
MASC Hipo ToM	5.87 ± 3.16	6.10 ± 2.49	0.204	0.808
MASC Hiper ToM	9.67 ± 4.12	9.50 ± 4.49	0.725	0.911
SCIP PD	61.93 ± 11.36	59.05 ± 12.00	0.785	0.482
SCIP Pc CL	42.27 ± 26.66	34.95 ± 26.60	0.450	0.432
PANSS P DS	17.87 ± 8.05	17.78 ± 9.83	0.271	0.978
PANSS N DS	22.87 ± 7.10	21.44 ± 7.81	0.959	0.591
PANSS C DS	−4.93 ± 5.36	−4.11 ± 5.03	0.807	0.653
PANSS PG PS	43.87 ± 19.47	42.50 ± 20.84	0.992	0.848

*Frequency of hits o successes (percentage).

**Figure 2:**
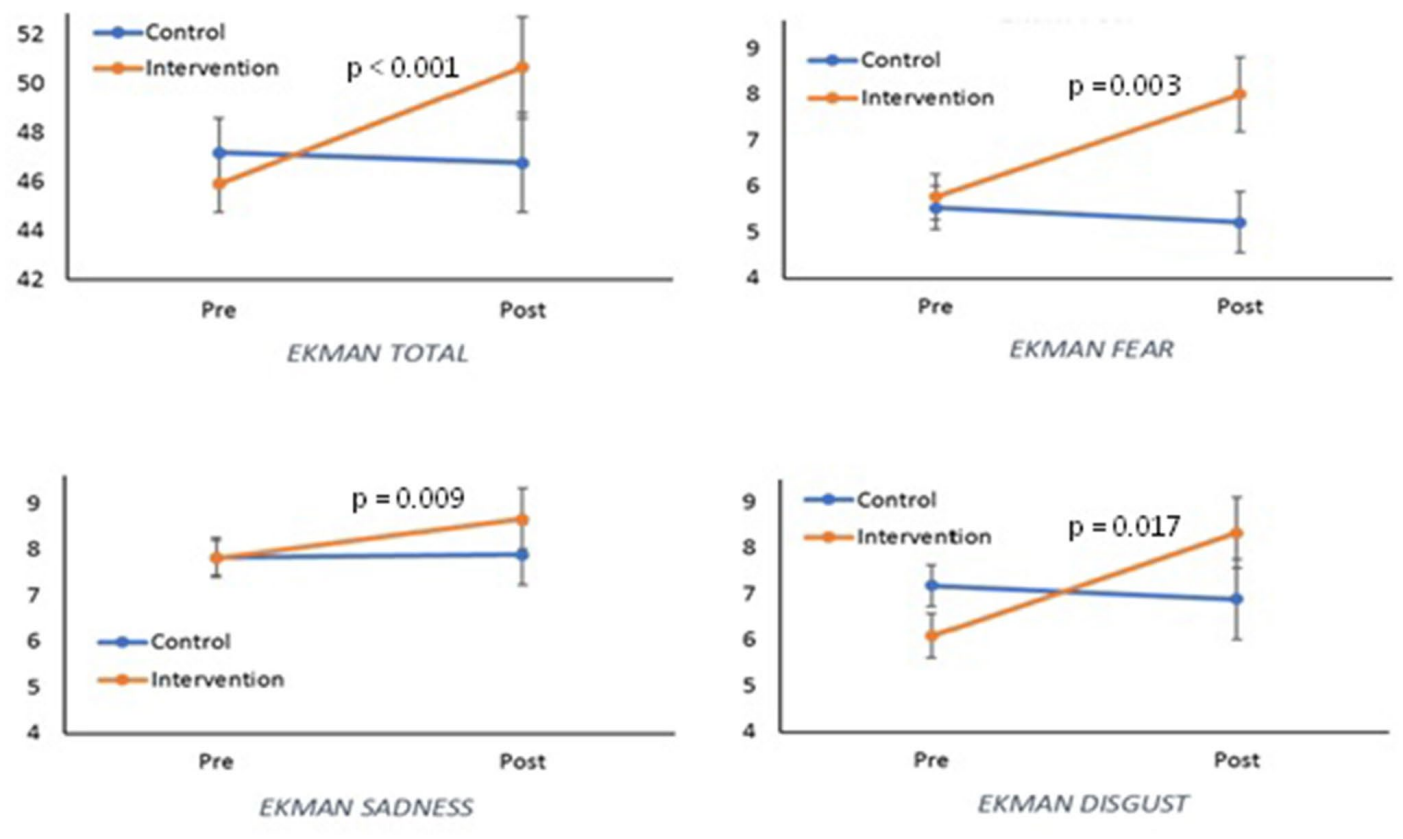
Evolution of the Ekman test scores and three of its components (fear, sadness and disgust) depending on the group to which they belong (*p*-value shows the significance of time x group interaction).

**Figure 3:**
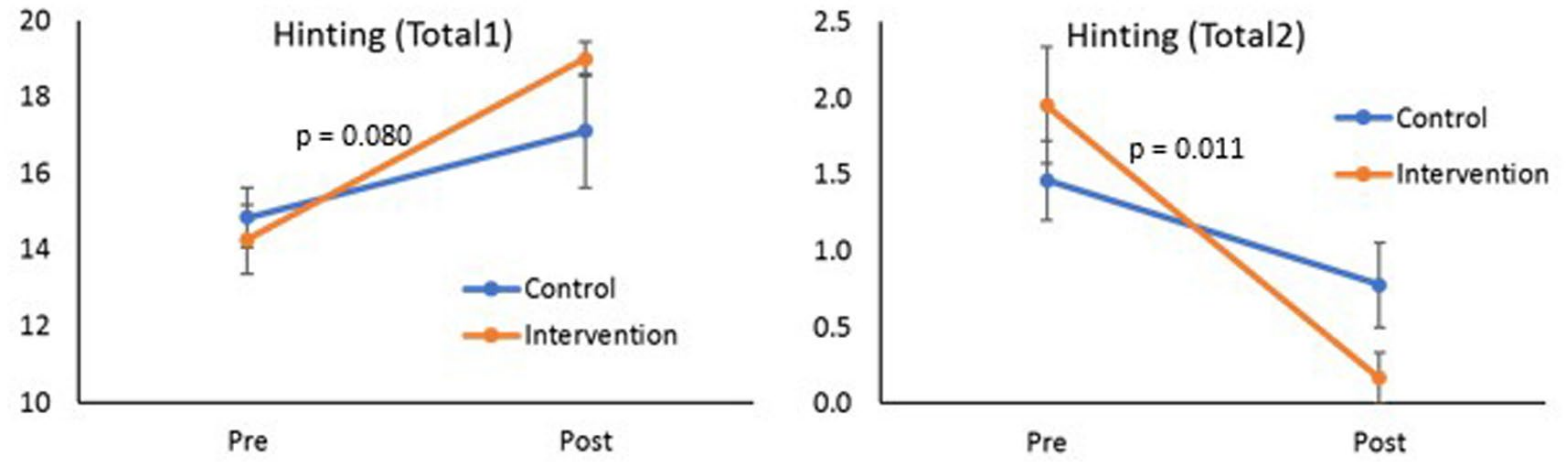
Evolution of the Hinting test scores (Total 1 and Total 2) depending on the group to which they belong (*p*-value shows the significance of time x group interaction).

Table 2 shows the comparison of the variables measured with the different tests between the pre and post values for each of the groups. In the last column of this table, the pre values between groups were also compared, not showing significant differences. In Ekman’s 60-item test, a significant improvement is perceived in the total score of the intervention group (*p* = 0.009), but not of the control group (*p* = 0.187), mainly due to the improvement in the emotions fear, sadness and disgust (*p* = 0.041, *p* = 0.021 and *p* = 0.038, respectively, Figure 2). An improvement in the Total 1 score (equivalently a reduction in the Total 2 score) of the Hinting test was also observed in both groups (control: *p* = 0.049, intervention: *p* < 0.001, Figure 3). In the Faux Pas FP-FP test, a similar effect was also shown, a greater improvement in the intervention group than in the control group (control: *p* = 0.046, intervention: p < 0.001). Finally, the Faux Pas C-C test shows a tendency toward improvement that is close to significance (*p* = 0.061). The comparison of whether the evolution over time of the different scores varied between the groups is shown in Table 3 (group*time column). Indeed, it showed a significant improvement in the intervention group compared to the control group (*p* < 0.001) in the Ekman test, due to a better performance on the emotion fear (*p* = 0.003), sadness (*p* = 0.009) and close to significance in disgust (*p* = 0.063). In the case of the Hinting test, a significant improvement (score reduction) in the Total 2 score is observed, being higher in the intervention group than in the control group (*p* = 0.011) and an improvement close to significance (*p* = 0.080), was higher in the Total 1 score of the intervention group. For the rest of the variables, no significant differences were found in the evolution of the two groups.

**Table 2:** Comparison within each of the groups between the pre and post values and comparison between pre values.

	Control Group			Intervention Group			Pre-Comparison	Effect size
	Pre (*N* = 28)	Post (*N* = 9)	*p* value	Pre (*N* = 22)	Post (*N* = 6)	*p* value	*p* value	
Ekman total	47.18 ± 7.60	46.78 ± 6.06	0.187	45.91 ± 5.42	50.67 ± 5.09	0.009	0.511	0.512
Ekman joy	9.57 ± 1.71	10	-	9.86 ± 0.35	10	-	0.435	-
Ekman fear	5.54 ± 2.52	5.22 ± 1.99	0.426	5.77 ± 2.35	8.00 ± 2.00	0.041	0.735	1.103
Ekman surprise	9.39 ± 0.83	9.33 ± 1.32	0.438	8.86 ± 2.17	7.50 ± 1.05	0.087	0.241	−2.205
Ekman sadness	7.82 ± 2.28	7.89 ± 1.96	0.684	7.82 ± 1.74	8.67 ± 1.63	0.021	0.996	0.342
Ekman disgust	7.18 ± 2.39	6.89 ± 2.62	0.790	6.09 ± 2.24	8.33 ± 1.86	0.038	0.107	0.603
Ekman anger	7.54 ± 2.08	7.44 ± 1.51	0.302	7.50 ± 1.68	8.17 ± 1.47	0.367	0.948	0.351
Hinting total 1	14.85 ± 4.01	17.11 ± 4.48	0.049	14.27 ± 4.24	19.00 ± 1.10	<0.001	0.633	0.471
Hinting total 2	1.46 ± 1.33	0.78 ± 0.83	0.001	1.95 ± 1.79	0.17 ± 0.41	<0.001	0.280	−0.459
Happé C	8.79 ± 2.99	9.89 ± 2.98	0.487	8.50 ± 2.42	9.33 ± 2.16	0.462	0.728	−0.187
Happé ToM	9.48 ± 3.98	10.89 ± 4.70	0.207	10.39 ± 2.17	11.83 ± 2.56	0.250	0.392	0.236
Faux pas FP-FP	33.30 ± 15.23	38.89 ± 14.53	0.046	32.17 ± 9.06	43.00 ± 13.18	0.001	0.785	0.270
Faux pas C-FP	17.25 ± 3.93	18.44 ± 1.88	0.290	17.72 ± 2.91	18.67 ± 0.98	0.455	0.679	0.059
Faux pas FP-C	18.40 ± 1.98	19.33 ± 1.66	0.226	18.67 ± 0.91	19.33 ± 0.82	0.151	0.604	0
Faux pas C-C	18.00 ± 1.69	18.33 ± 2.50	0.982	18.39 ± 1.24	19.33 ± 0.82	0.061	0.428	0.592
AIHQ HB	1.55 ± 0.21	1.62 ± 0.26	0.183	1.54 ± 0.26	1.74 ± 0.35	0.146	0.892	0.571
AIHQ IS	3.10 ± 0.82	3.13 ± 0.78	0.743	3.17 ± 0.77	2.96 ± 1.01	0.644	0.772	−0.207
AIHQ AS	2.43 ± 0.81	2.61 ± 0.75	0.531	2.62 ± 0.60	2.32 ± 0.76	0.297	0.426	−0.358
AIHQ BS	2.45 ± 0.75	2.72 ± 0.83	0.563	2.80 ± 0.59	2.52 ± 0.71	0.065	0.117	−0.267
AIHQ AB	1.48 ± 0.23	1.54 ± 0.44	0.633	1.51 ± 0.27	1.54 ± 0.36	0.618	0.704	0.000
MASC	23.63 ± 6.94	26.22 ± 5.61	0.256	26.31 ± 6.19	27.17 ± 7.68	0.076	0.240	0.137
MASC C*	13 (68.4%)	5 (55.6%)	0.775	10 (62.5%)	4 (66.7%)	0.856	0.723	-
MASC No ToM	5.11 ± 3.36	4.11 ± 3.02	0.188	3.94 ± 2.89	3.17 ± 2.32	0.103	0.283	−0.280
MASC Hipo ToM	6.21 ± 3.03	6.44 ± 2.83	0.636	5.75 ± 2.46	4.33 ± 2.73	0.123	0.629	−0.696
MASC Hiper ToM	10.05 ± 4.03	8.22 ± 3.77	0.301	9.00 ± 4.60	10.33 ± 5.35	0.974	0.476	0.524
SCIP PD	57.61 ± 10.61	58.22 ± 9.80	0.630	63.38 ± 12.31	70.33 ± 10.17	0.339	0.152	1.141
SCIP Pc CL	28.83 ± 23.62	31.56 ± 20.82	0.575	48.69 ± 26.22	71.83 ± 10.57	0.007	0.027	1.705
PANSS P PD	17.71 ± 9.78	19.56 ± 9.15	0.182	17.94 ± 8.23	14.80 ± 6.38	0.060	0.942	−0.487
PANSS N PD	22.18 ± 7.91	23.44 ± 5.94	0.474	22.00 ± 7.11	18.60 ± 4.77	0.941	0.947	−0.612
PANSS C PD	−4.41 ± 3.43	−3.22 ± 4.71	0.359	−4.56 ± 6.58	−3.80 ± 4.44	0.578	0.934	−0.169
PANSS G PD	44.82 ± 23.20	49.89 ± 21.42	0.448	41.31 ± 16.32	33.20 ± 7.40	0.454	0.621	−0.719

*Frequency of hits/successes (percentage).

**Table 3:** Comparison between the values of each group and time (pre or post) using mixed models.

	Control group		Intervention group		*p* value		
	Pre (*N* = 28)	Post (*N* = 9)	Pre (*N* = 22)	Post (*N* = 6)	Group	Time	Group*time
Ekman total	47.18 ± 7.60	46.78 ± 6.06	45.91 ± 5.42	50.67 ± 5.09	0.008	<0.001	<0.001
Ekman joy	9.57 ± 1.71	10	9.86 ± 0.35	10	-	-	-
Ekman fear	5.54 ± 2.52	5.22 ± 1.99	5.77 ± 2.35	8.00 ± 2.00	0.002	0.012	0.003
Ekman surprise	9.39 ± 0.83	9.33 ± 1.32	8.86 ± 2.17	7.50 ± 1.05	0.015	0.037	0.117
Ekman sadness	7.82 ± 2.28	7.89 ± 1.96	7.82 ± 1.74	8.67 ± 1.63	0.015	0.003	0.009
Ekman disgust	7.18 ± 2.39	6.89 ± 2.62	6.09 ± 2.24	8.33 ± 1.86	0.709	0.017	0.063
Ekman anger	7.54 ± 2.08	7.44 ± 1.51	7.50 ± 1.68	8.17 ± 1.47	0.252	0.272	0.248
Hinting total 1	14.85 ± 4.01	17.11 ± 4.48	14.27 ± 4.24	19.00 ± 1.10	0.117	0.001	0.080
Hinting total 2	1.46 ± 1.33	0.78 ± 0.83	1.95 ± 1.79	0.17 ± 0.41	0.026	<0.001	0.011
Happé C	8.79 ± 2.99	9.89 ± 2.98	8.50 ± 2.42	9.33 ± 2.16	0.899	0.431	0.928
Happé ToM	9.48 ± 3.98	10.89 ± 4.70	10.39 ± 2.17	11.83 ± 2.56	0.339	0.126	0.619
Faux pas FP-FP	33.30 ± 15.23	38.89 ± 14.53	32.17 ± 9.06	43.00 ± 13.18	0.951	0.022	0.855
Faux pas C-FP	17.25 ± 3.93	18.44 ± 1.88	17.72 ± 2.91	18.67 ± 0.98	0.787	0.454	0.832
Faux pas FP-C	18.40 ± 1.98	19.33 ± 1.66	18.67 ± 0.91	19.33 ± 0.82	0.986	0.318	0.792
Faux pas C-C	18.00 ± 1.69	18.33 ± 2.50	18.39 ± 1.24	19.33 ± 0.82	0.352	0.388	0.566
AIHQ HB	1.55 ± 0.21	1.62 ± 0.26	1.54 ± 0.26	1.74 ± 0.35	0.354	0.043	0.271
AIHQ IS	3.10 ± 0.82	3.13 ± 0.78	3.17 ± 0.77	2.96 ± 1.01	0.917	0.594	0.891
AIHQ AS	2.43 ± 0.81	2.61 ± 0.75	2.62 ± 0.60	2.32 ± 0.76	0.618	0.364	0.261
AIHQ BS	2.45 ± 0.75	2.72 ± 0.83	2.80 ± 0.59	2.52 ± 0.71	0.884	0.199	0.169
AIHQ AB	1.48 ± 0.23	1.54 ± 0.44	1.51 ± 0.27	1.54 ± 0.36	0.908	0.764	0.785
MASC	23.63 ± 6.94	26.22 ± 5.61	26.31 ± 6.19	27.17 ± 7.68	0.140	0.105	0.541
MASC C*	13 (68.4%)	5 (55.6%)	10 (62.5%)	4 (66.7%)	1.000		
MASC No ToM	5.11 ± 3.36	4.11 ± 3.02	3.94 ± 2.89	3.17 ± 2.32	0.221	0.126	0.804
MASC Hipo ToM	6.21 ± 3.03	6.44 ± 2.83	5.75 ± 2.46	4.33 ± 2.73	0.074	0.138	0.140
MASC Hiper ToM	10.05 ± 4.03	8.22 ± 3.77	9.00 ± 4.60	10.33 ± 5.35	0.950	0.926	0.452
SCIP (DS Direct score)	57.61 ± 10.61	58.22 ± 9.80	63.38 ± 12.31	70.33 ± 10.17	0.155	0.433	0.759
SCIP (PS percentile score)	28.83 ± 23.62	31.56 ± 20.82	48.69 ± 26.22	71.83 ± 10.57	0.003	0.039	0.208
PANSS P (DS)	17.71 ± 9.78	19.56 ± 9.15	17.94 ± 8.23	14.80 ± 6.38	0.678	0.036	0.030
PANSS N (DS)	22.18 ± 7.91	23.44 ± 5.94	22.00 ± 7.11	18.60 ± 4.77	0.932	0.913	0.718
PANSS C (DS)	−4.41 ± 3.43	−3.22 ± 4.71	−4.56 ± 6.58	−3.80 ± 4.44	0.796	0.671	0.766
PANSS G (PS)	44.82 ± 23.20	49.89 ± 21.42	41.31 ± 16.32	33.20 ± 7.40	0.732	0.543	0.462

*Frequency of hits/successes (percentage).

To measure the internal consistency (reliability) of all the pre and post variables, we performed the Cronbach’s alpha coefficient and obtained α = 0.522. In the case of the post variables, the Cronbach’s alpha value was 0.687.

## 4. Conclusion

Cognitive therapy in people with schizophrenia is primarily aimed at improving global social functioning (52). Social cognition programs can help the general functioning of people with psychosis, in addition neurocognition-based programs. Due to the need for a tool in Spanish for the rehabilitation of SC, a research team from the University of Santiago de Compostela designs the ET program showing its feasibility in a pilot study (29). Subsequently, this same research team conducted the first randomized controlled trial of this program, showing it to be a promising tool for treating SC deficits in schizophrenia (30).

In the Canary Islands, more specifically on the island of Tenerife, this study was replicated with a research team specialized in mental health in an attempt to accommodate the needs of this type of population. In conclusion, the results found in this study were very similar to those found by the Santiago team (30).

Recently, in 2021, Lahera et al. (53) compared in a group of 100 participants with schizophrenia the efficacy of using Training in Affect Recognition (TAR) and Social Cognition and Interaction Training (SCIT) on the performance of facial recognition of affect, ToM, AS and social functioning before, after treatment and 3 months later. They found that the entire sample, who received either TAR or SCIT, showed improvements in ToM, AS, clinical symptoms, and social functioning. However, the TAR intervention was more effective than the SCIT program in improving facial emotion recognition (ER).

With regard to the ER, the intervention group showed a significant improvement in the Ekman 60 Faces Test in the same way as in the Santiago research (30). These results are consistent with previous interventions (54, 55). Regarding the ToM, our intervention group showed significant improvements in the Hinting Task and Faux Pas Recognition Test as in the previous study by Maroño-Souto et al. (30). Evidencing in this way that both progresses did not reach the level of competence of the population without schizophrenia, which differs from those found in other previous interventions (56, 57).

In short, ET is an interactive online program that has proven to be effective for training the domains of social cognition. Compared to other programs available for SC rehabilitation (58), ET allows self-training and online follow-up by therapists. Thus becoming one of the intervention tools within the Spanish SMD community. The creation of this computerized tool enables a new approach where participants can practice social skills in a safe environment before facing society.

Our study has a series of limitations centered mainly on the sample size. Due to the COVID-19 pandemic, it was difficult to carry out an adequate follow-up of the study, as we have already mentioned, so the adequacy of the sample had to be reduced. In particular, the initial statistical power of 90% was reduced to 41.7%, which may be masking results that do not show significant differences between groups that do. On the other hand, all participants in the study at the time of the investigation were stabilized, which means that our results can be applied with caution to the general population with schizophrenia. 76% of the study participants were men, although the existence of sex difference in the risk of developing schizophrenia is well known (59), these percentages may vary depending on the bibliography consulted. The fact that the control group has not received any type of treatment, except that of the IPS, is also a limitation of this work.

Future lines of research on the topic should include more study about emotions and theory of mind in people with schizophrenia.

## Data availability statement

The original contributions presented in the study are included in the article/supplementary materials, further inquiries can be directed to the corresponding author.

## Ethics statement

The studies involving humans were approved by Research Ethics Committee University Hospital Complex of the Canary Islands. The studies were conducted in accordance with the local legislation and institutional requirements. The participants provided their written informed consent to participate in this study.

## Author contributions

FR (principal investigator), NC and EG designed, conducted and supervised the study and interpreted the results. EG also contributed to the statistical analyses. AG provided all standardised tests and supervised the design and execution of the study. MH, SL and PV searched for and selected participants. CL conducted the pre- and post-tests as a blinded researcher and assisted in data collection and extraction. FR, NC and EG drafted the manuscript. All authors have had full access to the study data.

## Footnotes

^1^www.e-motionaltraining.com
